# Efficacy and safety of complementary and alternative medicine therapy for gastroparesis: A protocol for systematic review and meta-analysis

**DOI:** 10.1097/MD.0000000000023294

**Published:** 2020-11-20

**Authors:** Manqiang Sun, Qi Chen, Quanwang Li, Lei Gao, Qin Zhou, Tian Zhou, Jianfeng Wang, Hua Duan, Haoyue Pang, Kaiwen Hu

**Affiliations:** a Dongfang hospital Beijing University of Chinese Medicine, No.6 Community 1 Fangxingyuan, Fengtai District; b Dongzhimen Hospital of Beijing University of Chinese Medicine, No. 5 Haiyuncang, Dongcheng District, Beijing, China.

**Keywords:** complementary and alternative medicine, gastroparesis, meta-analysis, systematic review

## Abstract

**Background:**

Gastroparesis affects the quality of life of many patients, but there is no effective treatment. Now, complementary and alternative medicine originated from China is gradually accepted by the world because of its unique treatment principles and relatively safe treatment methods. However, at present, there is still a lack of more definitive clinical application evidence for the treatment of gastroparesis with complementary and alternative medicine to confirm the safety and efficacy of complementary and alternative medicine in the treatment of gastroparesis caused by various causes. More comprehensive and stronger evidence-based medicine evidence is needed.

**Methods:**

We will retrieve literatures using Medline, Embase, the Cochrane Library database, Web of science, CNKI, VIP, CBM, and WanFang. We will look for RCTs or CCTs on the use of complementary and alternative medicine in the treatment of gastroparesis, and extract relevant data into the excel sheet. The whole retrieval and data extraction process were carried out by 2 researchers independently. Then we will use meta-analysis to make statistical analysis of all the results and make a systematic review of all the included literatures.

**Results:**

All results and safety data were analyzed for a comprehensive evaluation and/or descriptive analysis of the efficacy and safety of complementary and alternative therapies for gastroparesis.

**Conclusion:**

This study will provide more comprehensive clinical evidence for the treatment of gastroparesis with complementary and alternative therapies.

**Registration:**

The research has been registered and approved on the INPLASY.COM website. The registration number is INPLASY2020100033.

## 1 Introduction

Gastroparesis is a pathological condition characterized by delayed gastric emptying of solid food in the absence of mechanical obstruction of the stomach, which could result in some clinical signs and symptoms, such as early satiety, post-meal satiety, nausea, vomiting, burping, and bloating, upper abdominal discomfort or pain.^[1]^ Gastroparesis is difficult to distinguish from other digestive disorders, such as functional dyspepsia, because of these similar gastrointestinal symptoms, but gastroparesis is now recognized as a neuromuscular dysfunction of the stomach, including impaired gastric regulation.

The causes of gastroparesis currently include idiopathic diseases, diabetes mellitus, medical interventions or post-operative, neurological diseases, viral or bacterial infections, connective tissue disorders, or renal insufficiency.^[2]^ The principles of treatment for gastroparesis include correcting the deficiency or imbalance of electrolyte and nutrient, finding and treating the original cause of delayed gastric emptying, and treating specific symptoms. The treatment strategies currently rely mainly on dietary adjustments, withdrawal of medications that affect the normal function of the stomach, the use of antiemetic medications, and non-pharmacological measures such as endoscopic or surgical interventions or gastric electrical stimulation.^[3]^ However, in clinical practice, accelerating or normalizing gastric emptying may not improve patients’ clinical symptoms, and many patients often do not respond to pharmacological treatments. Current alternative non-drug therapy strategies, such as endoscopy, electrical stimulation, or surgery, are mainly used in patients with severe gastroparesis. Therefore, it is very important to seek effective complementary and alternative therapies that can be widely used to relieve the suffering of patients with gastroparesis.

There have been a number of clinical trials reporting the effectiveness of complementary and alternative therapies for gastroparesis, particularly in China. These treatments (such as acupuncture and moxibustion) can relieve gastrointestinal symptoms and improve patients’ quality of life. But higher levels of evidence to confirm the efficacy and safety of complementary and alternative therapies are still lacking, which makes the treatment of gastroparesis more complicated and difficult.

## 2 Review aims

The purpose of this meta-analysis was to review complementary and alternative therapies available for the treatment of gastroparesis through a systematic and extensive search. We plan to further clarify the effectiveness of complementary and alternative therapies in the treatment of gastroparesis by summarizing and analyzing existing clinical evidence. At the same time, we will further summarize the safety of complementary and alternative therapies through systematic review and meta-analysis, so as to provide more useful guidance for later clinical practice.

## 3 Methods

### 3.1 Protocol and registration

The protocol has been registered and approved on the PROSPERO website (registration number: INPLASY2020100033), which is available at doi: 10.37766/inplasy2020.10.0033. This research will follow the guidelines of the preferred reporting items for systematic reviews and meta-analyses (PRISMA).^[4]^ This systematic review does not require ethical approval because the data are based on the published databases.

### 3.2 Eligibility criteria

#### 3.2.1 Type of participants

Patients with a diagnosis of gastroparesis. Patients with postprandial epigastric discomfort (such as nausea, vomiting, postprandial fullness, abdominal distension, and epigastric pain) and with delayed gastric emptying or dysregulation of the stomach as assessed by the gastric exercise test. We excluded those patients whose upper gastrointestinal endoscopy revealed significant pathological changes.

#### 3.2.2 Interventions

We include all complementary and alternative therapies that exclude oral and surgical treatments which have been shown to have no significant therapeutic effect or to pose a significant safety risk. Now known complementary and alternative therapies may include acupuncture, moxibustion, acupoint application, and so on.

#### 3.2.3 Type of Controls

The control group can be neither on-treatment nor standard treatment, as long as it does not receive specific complementary and alternative therapy corresponding to the experimental group.

#### 3.2.4 Outcomes

We focused on assessing the symptoms of gastroparesis reported by patients in the current clinical study, including the Gastroparesis Symptom Index (GCSI), the Comprehensive Patient Assessment of symptoms of Upper gastrointestinal disease (PAGI-SYMP), and a revised GCSI-DAILY Diary (GCSI-DD). We also assessed changes in patients quality of life (through PAGI-QOL), and psychosomatic changes (through BDI and STAI). All treatment-related adverse events will be recorded, summarized, and evaluated.

#### 3.2.5 Type of study designs

We only include Randomized controlled trials (RCTs) or controlled clinical trials (CCTs), which aimed to evaluate the efficacy of complementary and alternative therapies in the treatment of gastroparesis.

### 3.3 Data source and search strategy

We will retrieve literature using the following data sources: Medline (through PubMed), Embase, the Cochrane Library database (Cochrane Central Register of Controlled Trials), Web of science, as well as 4 Chinese databases (China National Knowledge Infrastructure, VIP Database for Chinese Technical Periodicals, Chinese Biomedical Literature Database, and WanFang). Other resources will be searched to make up for the deficiency of the electronic database, mainly on the corresponding website for clinical trial registration and grey literature on gastroplegia treatment. The retrieval policy details in Medline are shown in Table 1. We will make the necessary changes and equivalent translations of the search terms to ensure that all databases use the same search terms.

**Table 1:** Search strategy for the Medline database.

Number	Search terms
#1	(gastroparesis or gastroparesis syndrome or delayed gastric emptying or functional stomach evacuating disturbance or stomach palsy or gastroparesis or gastroparalysis or stomach paralysis or gastric paralysis or gastroatonia or gastroplegia). ti, ab;
#2	(acupuncture or traditional Chinese medicine or TCM or herb or electric or herbal or vitamin or probiotics or massage or electroacupuncture or STW 5 or Iberogast or Rikkunshito or Simotang or Taraxacum officinale or Dai-kenchu-to or Modified Xiaoyao San or Banxiaxiexin decoction or Marijuana or Cannibis sativa or endocannabinoids or anandamide or 2-arachidonylglycerol or cannabinoids or natural remedies or naturopath^∗^ or nutritional therapy or oriental medicine or weed or complementary alternative medicine or CAM).). ti, ab
#3	(controlled clinical trial or randomized or randomised or randomized controlled trial or randomised controlled trial). ti, ab;
#4	1 and 2 and 3

### 3.4 Selection criteria

**Figure 1:**
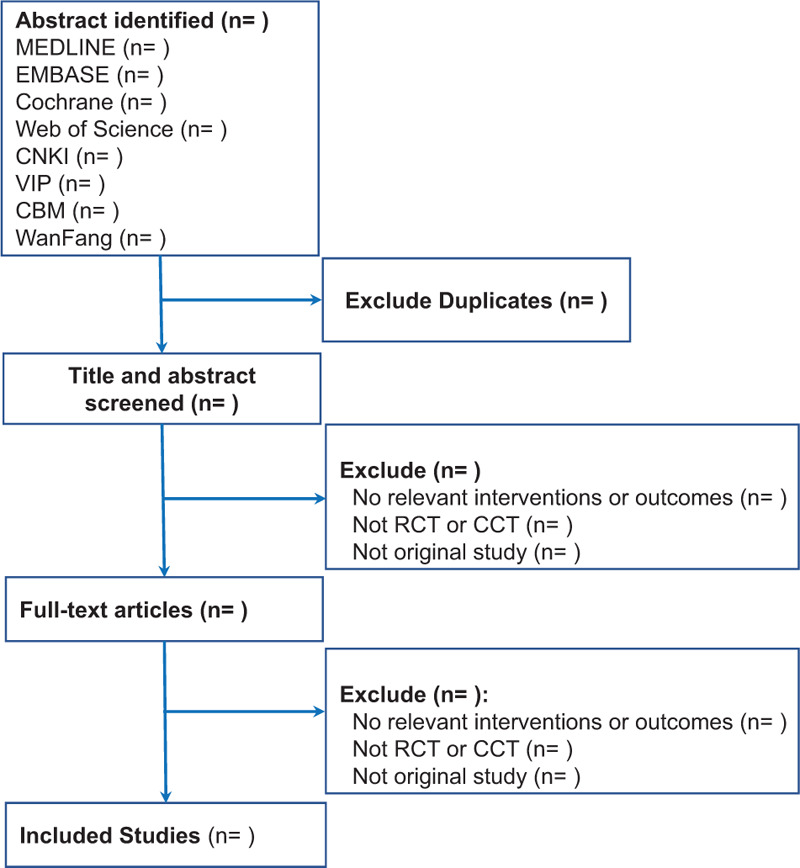
QUOROM flow diagram.

We will use EndNote X9 (USA) to manage all the retrieved documents. First, duplicate literature from different databases was excluded, and then that literature that was irrelevant to this study was excluded by reading the titles and abstracts of the literature, and then full-text reading and screening were conducted according to the pre-determined inclusion and exclusion criteria. This process will be independently reviewed and screened by 2 investigators. Any differences will be discussed between the 2 reviewers, and further differences will be arbitrated by the third author. Preferred reporting items for systematic reviews and meta-analysis flow diagram of literature screening and selection processes are shown in Figure 1.

### 3.5 Data extraction and quality assessment

After the unified inclusion literature is determined, the 2 researchers will independently extract the data based on a pre-established Excel sheet. The spreadsheet mainly includes the study title, author names, publication date, inclusion/exclusion criteria, the number of participants, the patients baseline characteristics (such as age, gender, degree of gastroplegia), follow-up time, the number of the experimental group and the type of intervention, the number of the control group and the type of intervention, the end-point indicators, and the changes in the variation of all indicators in follow-up time. When the article did not provide quantitative data that are needed we use Engauge Digitizer software to extract the exact figures from published figures. Any differences will be discussed between the 2 investigators and further differences will be arbitrated by the third author.

The deviation risk Cochrane collaboration tools (http://methods.cochrane.org/bias/assessing-risk-bias-include - studies) will be used to evaluate all potential sources of relative deviation. We summarize the individual and overall bias risk data included in the study. The main evaluation areas include random sequence generation; Allocation hiding; Blind testing of patients, researchers, and outcome assessors; Bias in reporting; Frayed prejudices; And other possible sources of deviation, such as those related to trial design, contamination risks, or cross-risks between the 2 groups.

### 3.6 Outcome estimation and Data Synthesis

Meta-analysis was used to summarize treatment outcomes. For dichotomous results, the results are expressed as a 95% confidence interval (CI) risk ratio (RR). For continuous variables, we will use the weighted mean difference (WMD) or standardized mean difference (SMD). If a result measure contains less than 2 trials, we will summarize the results descriptively. We will use *I*^2^ statistical analysis to estimate the percentage of variability due to non-random heterogeneity in the study. We will use the following rules to classify the heterogeneity. A value of 0% to 25% for *I*^2^ indicates low heterogeneity. A value of *I*^2^ between 25% and 50% indicates moderate heterogeneity. A value of *I*^2^ between 75% and 100% indicates high heterogeneity. When the heterogeneity of the results is low, the fixed-effect model will be used for the meta-analysis analysis. The RevMan 5.0.16 (Nordic Cochrane Centre, Cochrane Collaboration) and STATA 14.0 (STATA Corp LP) was used for statistical analysis.

### 3.7 Subgroup analysis

When aggregated results show significant heterogeneity, we first use subgroup analysis to find the source of heterogeneity. The pre-set grouping mainly includes age, gender, different intervention methods, different control methods, treatment time, patients grade of gastroparesis, combined with other diseases, and the quality of the study.

### 3.8 Sensitivity analysis

Each study included in the results will be excluded one by one, then the remaining study data will be re-analyzed and pooled, and the differences between the re-obtained effect and the original effect will be compared to test the stability of the results. The entire process is performed using STATA 14.0 software.

### 3.9 Publication bias

Results from more than 10 studies in the meta-analysis will be evaluated using the symmetry of the funnel plot and the Eggers test will be used to examine publication bias.

### 3.10 Grading the quality of evidence

In this systematic review, the quality of evidence throughout the study was assessed using the “Grades of Recommendations Assessment, Development and Evaluation (GRADE)” established by the World Health Organization and international organizations, which divides the quality of evidence into 4 levels: high, medium, low, and very low.

## 4 Discussion

According to American epidemiological statistics, the 10-year prevalence of gastroparesis is estimated at 37.8 per 100,000 people per year.^[5]^ Yet one study estimates that as many as 1.8% of the general population may have gastroparesis, but only 0.2% are diagnosed.^[6]^ Therefore, the actual incidence of gastroparesis may be much higher than the current epidemiological statistics. The risk factors for gastroparesis are unknown and may be related to gender, overweight or obesity, diabetes, smoking, or alcohol consumption.^[7–10]^ At present, although some progress has been made in the mechanism and pathophysiology of gastroparesis, there is still a great gap in knowledge and great inconsistency and heterogeneity among different studies. Therefore, it is the best research direction to study gastroparesis from the perspective of individual therapy rather than from the perspective of cellular mechanism.^[11]^ It is very important to realize that the gastrointestinal regulation of patients with gastroparesis is reduced, which is also a difficult problem to be solved by current precision therapy.

Gastroparesis and impaired gastric regulation function caused by gastric neuromuscular dysfunction has always been the advantages of traditional Chinese medicine which is characterized by a holistic view and individual treatment based on dialectical theory. In our previous prospective, multicenter, randomized, double-blind, parallel placebo-controlled clinical study,^[12]^ we found that the clinical effectiveness of gastric paralysis external party (a topical sticking method) in the treatment of postoperative gastroparesis syndrome can reach 68.3%, and gastric paralysis external party significantly reduce the symptoms of fullness in patients with Gastroparesis. In addition, only 5.6% of patients developed local itchy skin symptoms, but the symptoms disappeared after the removal of the application.

Acupuncture and moxibustion have been widely used in the treatment of symptomatic gastroparesis in China and some clinical studies have reported the effect of acupuncture and moxibustion in the treatment of gastroparesis. However, a recent Cochrane meta-analysis of acupuncture for symptomatic gastroparesis concluded no effect of acupuncture on gastroparesis was found and the safety data were lacking.^[13]^ These conflicting results make the clinical efficacy of complementary and alternative medicine in the treatment of gastroparesis very uncertain, so it is important to reintegrate the evidence for a systematic review.

At present, more and more countries and regions around the world begin to accept and use complementary and alternative medicines, which means that further systematic evaluation and meta-analysis are necessary to evaluate the effectiveness and safety of such interventions in patients with gastroparesis by combining all the evidence. This systematic review will provide the latest evidence of complementary and alternative therapies for gastroparesis, with particular attention to their efficacy and safety in improving gastric dysfunction and alleviating unpleasant symptoms for patients with gastroparesis.

## Author contributions

**Conceptualization:** Manqiang Sun, Qi Chen, Kaiwen Hu.

**Data curation:** Lei Gao, Hua Duan.

**Funding acquisition:** Kaiwen Hu.

**Methodology:** Tian Zhou, Quanwang Li.

**Project administration:** Jianfeng Wang.

**Resources:** Haoyue Pang.

**Supervision:** Qin Zhou.

## References

[1] Camilleri M Parkman HP Shafi MA Clinical guideline: management of gastroparesis. Am J Gastroenterol 2013;108:18–37. quiz 38. 23147521 10.1038/ajg.2012.373 PMC3722580

[2] Mearin F Pérez-Oliveras M Perelló A Dyspepsia and irritable bowel syndrome after a salmonella gastroenteritis outbreak: one-year follow-up cohort study. Gastroenterology 2005;129:98–104. 16012939 10.1053/j.gastro.2005.04.012

[3] Camilleri M Chedid V Ford AC Gastroparesis. Nat Rev Dis Primers 2018;4:41. 30385743 10.1038/s41572-018-0038-z

[4] Moher D Liberati A Tetzlaff J Preferred reporting items for systematic reviews and meta-analyses: the PRISMA statement. BMJ 2009;339:b2535. 19622551 10.1136/bmj.b2535 PMC2714657

[5] Jung HK Choung RS Locke GR 3rd The incidence, prevalence, and outcomes of patients with gastroparesis in Olmsted County, Minnesota, from 1996 to 2006. Gastroenterology 2009;136:1225–33. 19249393 10.1053/j.gastro.2008.12.047 PMC2705939

[6] Rey E Choung RS Schleck CD Prevalence of hidden gastroparesis in the community: the gastroparesis “iceberg”. J Neurogastroenterol Motil 2012;18:34–42. 22323986 10.5056/jnm.2012.18.1.34 PMC3271251

[7] Stanghellini V Tack J Gastroparesis: separate entity or just a part of dyspepsia? Gut 2014;63:1972–8. 25260920 10.1136/gutjnl-2013-306084

[8] Ravella K Al-Hendy A Sharan C Chronic estrogen deficiency causes gastroparesis by altering neuronal nitric oxide synthase function. Dig Dis Sci 2013;58:1507–15. 23504347 10.1007/s10620-013-2610-4 PMC3691310

[9] Showkat Ali M Tiscareno-Grejada I Locovei S Gender and estradiol as major factors in the expression and dimerization of nNOSα in rats with experimental diabetic gastroparesis. Dig Dis Sci 2012;57:2814–25. 22684582 10.1007/s10620-012-2230-4

[10] Parkman HP Yates K Hasler WL Clinical features of idiopathic gastroparesis vary with sex, body mass, symptom onset, delay in gastric emptying, and gastroparesis severity. Gastroenterology 2011;140:101–15. 20965184 10.1053/j.gastro.2010.10.015 PMC3089423

[11] Usai-Satta P Bellini M Morelli O Gastroparesis: new insights into an old disease. World J Gastroenterol 2020;26:2333–48. 32476797 10.3748/wjg.v26.i19.2333 PMC7243643

[12] Zhou Q Zuo M Tian Y Multi-centric clinical study of the effect of intervention time on efficacy of gastroparesis external application prescription treatment of gastrointestinal tumor postsurgical gastroparesis. Journal of Traditional Chinese Medical Sciences 2016;3:212–9.

[13] Kim KH Lee MS Choi TY Acupuncture for symptomatic gastroparesis. Cochrane Database Syst Rev 2018;12:Cd009676. 30560568 10.1002/14651858.CD009676.pub2 PMC6516818

